# Nitric Oxide-cGMP Signaling Stimulates Erythropoiesis through Multiple Lineage-Specific Transcription Factors: Clinical Implications and a Novel Target for Erythropoiesis

**DOI:** 10.1371/journal.pone.0144561

**Published:** 2016-01-04

**Authors:** Tohru Ikuta, Hassan Sellak, Nadine Odo, Adekunle D. Adekile, Karin M. L. Gaensler

**Affiliations:** 1 Department of Anesthesiology and Perioperative Medicine, Medical College of Georgia, Georgia Regents University, Augusta, Georgia, United States of America; 2 Department of Paediatrics, Faculty of Medicine, Kuwait University, Safat, Kuwait; 3 Division of Hematology/Oncology, Department of Medicine, University of California San Francisco, San Francisco, California, United States of America; Center for Cancer Research, National Cancer Institute, UNITED STATES

## Abstract

Much attention has been directed to the physiological effects of nitric oxide (NO)-cGMP signaling, but virtually nothing is known about its hematologic effects. We reported for the first time that cGMP signaling induces human γ-globin gene expression. Aiming at developing novel therapeutics for anemia, we examined here the hematologic effects of NO-cGMP signaling *in vivo* and *in vitro*. We treated wild-type mice with NO to activate soluble guanylate cyclase (sGC), a key enzyme of cGMP signaling. Compared to untreated mice, NO-treated mice had higher red blood cell counts and total hemoglobin but reduced leukocyte counts, demonstrating that when activated, NO-cGMP signaling exerts hematopoietic effects on multiple types of blood cells *in vivo*. We next generated mice which overexpressed rat sGC in erythroid and myeloid cells. The forced expression of sGCs activated cGMP signaling in both lineage cells. Compared with non-transgenic littermates, sGC mice exhibited hematologic changes similar to those of NO-treated mice. Consistently, a membrane-permeable cGMP enhanced the differentiation of hematopoietic progenitors toward erythroid-lineage cells but inhibited them toward myeloid-lineage cells by controlling multiple lineage-specific transcription factors. Human γ-globin gene expression was induced at low but appreciable levels in sGC mice carrying the human β-globin locus. Together, these results demonstrate that NO-cGMP signaling is capable of stimulating erythropoiesis in both *in vitro* and vivo settings by controlling the expression of multiple lineage-specific transcription factors, suggesting that cGMP signaling upregulates erythropoiesis at the level of gene transcription. The NO-cGMP signaling axis may constitute a novel target to stimulate erythropoiesis *in vivo*.

## Introduction

Nitric oxide (NO) plays a critical role in the regulation of vascular tone [1,2] and has anti-platelet [3] and anti-inflammatory properties [4]. NO availability is reduced in multiple clinical disorders including chronic heart disease [5], metabolic syndrome [6], and anemic disorders such as sickle cell disease (SCD) [7]. SCD is characterized by sickle hemoglobin polymerization, intravascular hemolysis, and low NO availability [8]. Our laboratory has demonstrated the therapeutic potential of NO in this disorder [9,10,11,12]. Interestingly, NO may have a role in the molecular actions of hydroxyurea (HU), a drug approved to treat SCD [13]. In SCD, HU increases fetal hemoglobin (HbF) synthesis, reduces the frequency of vaso-occlusive crisis and acute chest syndrome, and decreases blood transfusion needs [14,15]. Because NO is a product of HU metabolism [16,17], it is plausible that the reduction in vaso-occlusive crisis frequency among patients treated with HU can be attributed to NO [18]. NO activates cGMP-dependent pathways by activating soluble guanylate cyclase (sGC), an obligate heterodimer of an α-subunit (sGCα) and a β-subunit (sGCβ) [19]. In vitro studies from our laboratory and others showed that HU induces HbF expression by activating the sGC-cGMP signaling pathway [20,21,22]. HU was subsequently shown to act as an NO donor [23] and to activate cGMP signaling in the blood cells of SCD patients [24,25]. These lines of evidence suggest that NO-cGMP signaling contributes to the mechanisms of action of HU, however, the hematological effects of NO-cGMP signaling have not been studied in an in vivo setting.

This study was undertaken to test our hypothesis that if HU exerts hematological effects on blood cells at least in part through NO-cGMP signaling, then mice in whose blood cells NO-cGMP signaling was activated should demonstrate hematological changes similar to those of SCD patients treated with HU. We activated cGMP signaling in mouse blood cells by (1) treating mouse blood cells with low-dose NO, an sGC activator [26], and (2) generating mice which express rat sGC subunits at high levels in erythroid and myeloid cells. To verify that activating cGMP signaling induces HbF expression in erythroid cells [21], we bred sGC transgenic mice with a mouse carrying a yeast artificial chromosome (YAC) clone which includes the human β-globin gene locus [27]. We then examined the effect of cGMP signaling on the expression of lineage-specific transcription factors. To our knowledge, this study is the first to demonstrate the hematologic effects of NO-cGMP signaling in vivo. Our findings on the role of NO-cGMP signaling in hematopoiesis may lead to explanations for clinical observations in anemic disorders as well as to possible therapies.

## Materials and Methods

### Inhalation of low-dose NO and hematological analysis

Wild-type mice (C57BL/6, body weight, >25 g) were housed in a viral-free environment in standard approved chambers (5 mice/cage). Mice were allowed to breathe 8 parts per million (ppm) NO gas for 8 weeks as described [10]. To determine expression levels of the transgenes or investigate the effects of cGMP signaling on mouse blood cells, mice were anesthetized with ketamine/xylazine (0.1mg/0.015mg/g, IP) and euthanasia was performed by cervical dislocation with anesthesia. Blood was obtained from tail veins and bone marrow (BM) cells were harvested from femur bones. cGMP levels of red blood cells (RBCs) were measured using an ELISA kit (Cayman Laboratories, Ann Arbor, MI, USA) as described [28]. Complete blood count was performed using an automatic blood cell analyzer (Coulter A^c^•T diff analyzer, Beckman Coulter, Fullerton, CA, USA). All animal studies were approved by the Institutional Animal Care and Use Committees of the Georgia Regents University and the University of California San Francisco.

### Mouse BM cell analysis by flow cytometry, semi-solid colony assays, and immunoblotting

Cell surface antigens of mouse BM mononuclear cells (BMMNC) obtained from mice treated or not treated with 8 ppm NO were analyzed by flow cytometry (Becton-Dickinson FACScan, Franklin Lakes, NJ, USA) as described [29]. Fluorescein isothiocyanate or phycoerythrin (PE)-labeled antibodies were purchased from BD Biosciences (San Jose, CA, USA). Semi-solid cultures were performed using murine BM cells prepared from NO-treated mice, sGC transgenic mice, and human BM cells as described [30]. Following injections of phenylhydrazine, erythroblast-rich cells were prepared from the spleen [27] and isolated using magnetic-activated cell sorting columns (MidiMACS separator and Anti-Ter119 microbeads, Miltenyi Biotec Inc., Auburn, CA, USA) [28]. Mouse globin, glyceraldehyde 3-phosphate dehydrogenase (GAPDH), and vasodilator-stimulated phosphoprotein (VASP) expression in spleen-derived erythroblasts were determined by immunoblotting as described [31]. Antibodies used were: anti-mouse β-globin and anti-GAPDH (sc-31116 and sc-25778, Santa Cruz Biotechnology, Santa Cruz, CA, USA) and anti-phosphoVASP (Ser239) (#3114, Cell Signaling Technology, Danvers, MA, USA).

### Expression of lineage-specific transcription factors in murine and human BM progenitors treated with 8-bromo-cGMP

Expression levels of lineage-specific transcription factors in murine and human blood cells were determined by real time (RT)-PCR as described below. Murine erythroid cells were isolated from spleens as described above and leukocytes were isolated by density gradient centrifugation using Histopaque 1083 (Sigma Chemicals, St. Louis, MO, USA). BMMNCs from normal subjects were obtained from Poietics (Walkersville, MD, USA) and human CD34^+^ cells were provided by NHLBI PEGT Hematopoietic Cell Processing Core (Fred Hutchinson Cancer Research Center, Seattle, WA, USA). Human erythroid and myeloid cells were isolated from semi-solid cultures in which CD34^+^ cells (4 × 10^5^ cells) were plated in the presence of 8-bromo-cGMP as described [28]. Total RNA was extracted from individual colonies (RNeasy Mini Kit, Qiagen) and cDNA was generated (SuperScript II Reverse Transcriptase kit, Invitrogen). RT-PCR was carried out with the Mx3000p System (StrataGen) using iQ SYBR Green Supermix (Bio-Rad) according to the manufacturer’s instructions. All amplifications were performed in triplicate and 18S rRNA was used as the internal control. Relative expression was quantitated using the standard Δ/ΔC_t_ method. Primer sequences are available in S1 Text.

### Generation of sGC transgenic mice and sGC activity in murine blood cells

To confirm that the hematologic effects of cGMP signaling were unaffected by mouse genetic backgrounds, we established four transgenic lines in B6CBA. Two DNA fragments containing cDNA for sGCα or sGCβ driven by the mini β-locus control region (βLCR) and a β-globin gene promoter were injected into fertilized eggs and four founder mice carrying both sGCα and sGCβ were identified. Detailed procedures for determining copy number of the transgenes (S1 Table) and transgene expression are described in S1 Text. To generate sGC-YAC mice, hemizygous male sGC mice were bred with homozygous female YAC mice carrying a YAC clone (βYAC) [27]. To analyze the level of human globin chains in sGC/YAC mice, mice doubly hemizygous for rat sGC (sGCα/β) and the βYAC transgene were compared with littermates carrying the βYAC but not sGC transgenes. To determine sGC activity, erythroblast-rich cells were isolated from the spleen as above and peripheral leukocytes were isolated by density gradient centrifugation (Histopaque 1083, Sigma Chemicals). See S1 Text for detailed procedures.

### Globin chain analysis by high performance liquid chromatography

Peripheral blood was isolated from fetal and adult mice and hemolysates were prepared. Hemolysates were analyzed using a Shimadzu LC-VP series system (Shimadzu, Kyoto, Japan) and a Vydac C4 column (250 × 4.6 mm), as described [28].

### Chromatin immunoprecipitation (ChIP) assays

ChIP assays were performed as described [32]. Briefly, genomic DNA was cross-linked using 1% formaldehyde and extracted by standard procedures. After cleaning with salmon sperm DNA/protein A agarose-50% slurry, RNA pol II antibody (EMDMillipore, Billerica, MA, USA) was added and the immune complexes were subjected to digestion with 20 mg/mL proteinase K. Immunoprecipitated chromatins were then purified (Qiaquick PCR purification kits, Qiagen). PCR was performed using primers for the β and γ-globin core promoter sequences [33]. PCR products were separated in agarose gels and band intensities were quantitated (Image J 1.47, NIH). Procedure details and primer sequences can be found in S1 Text.

### Statistical analysis

All experiments were performed at least in triplicate and data are shown as mean ± standard error of mean. Student’s t-test or Mann-Whitney test was performed to compare hematologic parameters between animal groups. *P* values of less than 0.05 were considered statistically significant.

## Results

### *In vivo* and *in vitro* studies of hematopoietic effects of NO

**Fig 1:**
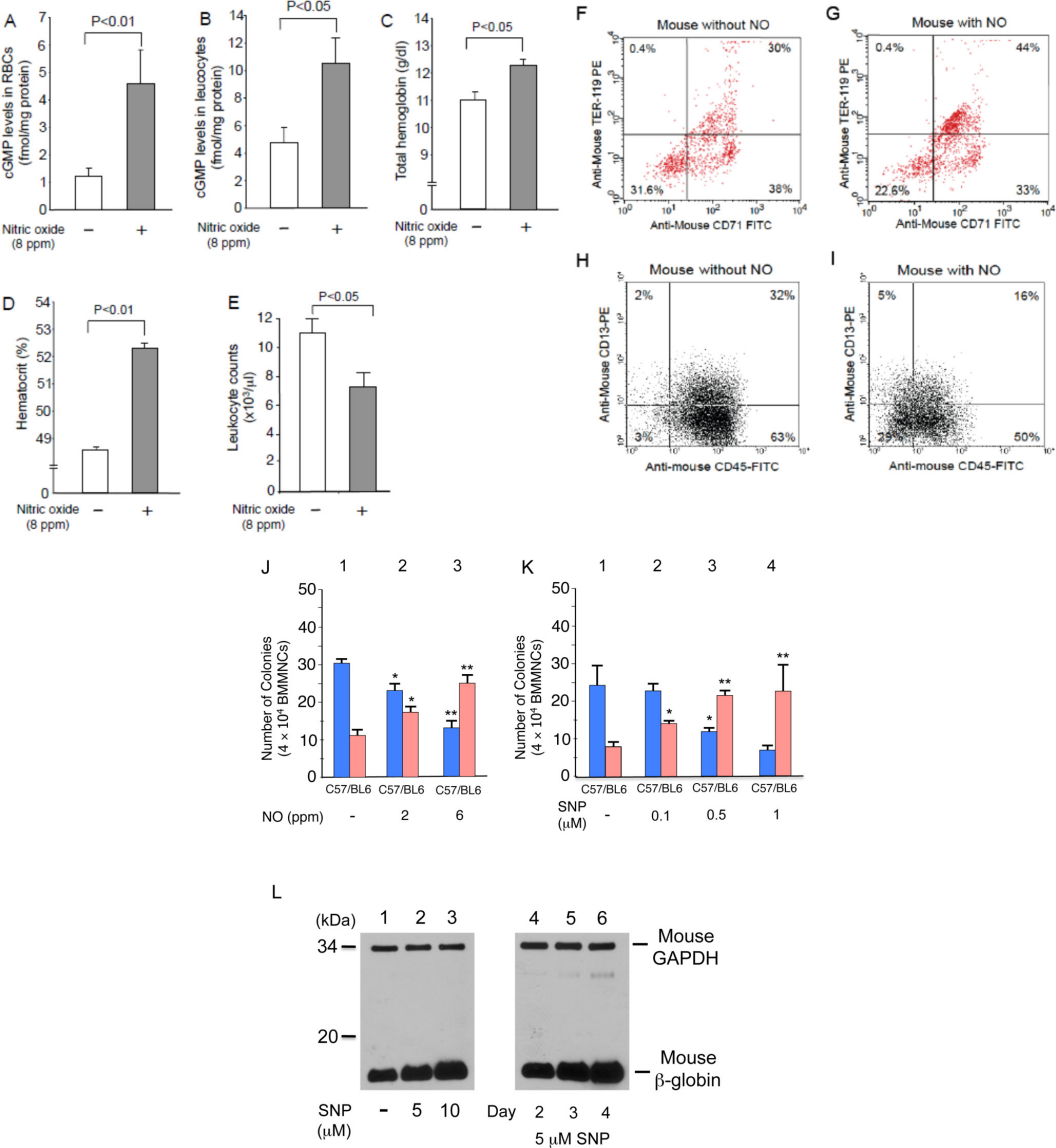
NO-cGMP signaling modulates hematopoiesis *in vivo*. **(A-E)** NO upregulates erythropoiesis but suppresses myelopoiesis. Wild-type mice (n = 4) were treated with 8 ppm NO for 8 weeks. Intracellular cGMP levels of RBCs (A), peripheral blood leukocytes (B), total hemoglobin levels (C), hematocrit (D), and leukocyte count (E) were determined. P values are shown above the figures. **(F-I)** NO affects cell-lineage populations in BM. Mouse BMMNC, with or without 8 weeks of NO inhalation treatment, were examined for CD antigens. Results are shown for erythroid-lineage cells (F&G) and leukocytes (H&I). Experiments were repeated 3 times using BMMNCs from 3 defferent mice and representative results are shown. **(J&K)** NO affects cell differentiation of hematopoietic progenitors. Semi-solid colony assays were performed with BMMNC prepared from mice treated with 2 or 6 ppm NO for 8 weeks (J). The cells were plated on semi-solid plates with various SNP concentrations (K). The numbers of erythroid- (pink) and myeloid-lineage (blue) cells are shown. **(L)** NO donor upregulates βb-globin expression. Murine globin expression in erythroblasts prepared from semi-solid cultures was analyzed by immunoblotting. Cellular extracts (10 μg) were loaded to gels and murine βb-globin was detected by antibody.

In wild-type mice treated for 2 months with 8 ppm NO [10], we found that the intracellular cGMP levels of RBCs and leukocytes were elevated 2- to 3-fold (Fig 1A & 1B, *P*<0.01&*P*<0.05), an indication that cGMP signaling had been activated. Interestingly, NO-treated wild-type mice had higher total hemoglobin (Fig 1C, P<0.02) and hematocrit (Fig 1D, P<0.05), but significantly reduced leukocyte counts (Fig 1E, P<0.01). To determine whether NO modulated the differentiation of hematopoietic progenitors, we used flow cytometry to analyze the erythroid and myeloid cell populations in the BM of mice treated with 8 ppm NO (Fig 1F to 1I). Erythroid cells, defined as those positive for TER 119 and CD71, increased from 30% to 44% (Fig 1F & 1G), while the number of myeloid cells stained by CD13 and CD45 were halved from 32% to 16% (Fig 1H & 1I). To confirm the effect of NO on progenitor cell differentiation, we performed semi-solid colony assays using BM cells isolated from NO-treated mice (Fig 1J); here we treated mice with 2 to 6 ppm NO gas as 8 ppm NO gas showed the strongest hematologic effects but sometimes toxic and administering 2 to 6 ppm NO gas was sufficient to study the hematologic effects of NO. NO breathing reduced the number of myeloid colonies but increased erythroid colonies in a dose responsive manner. Similar results were obtained with semi-solid colony assays using the NO donor sodium nitroprusside (SNP) (Fig 1K). Consistently, the expression of mouse β-globin was increased in erythroblasts isolated from semi-solid cultures treated with SNP (Fig 1L). This supports the notion that NO inhalation increases total hemoglobin levels in mice (Fig 1C). Thus, NO may stimulate the differentiation of hematopoietic progenitors to erythroid-lineage cells but suppress it to myeloid-lineage cells.

### Generation and characterization of mice overexpressing rat sGC

**Fig 2:**
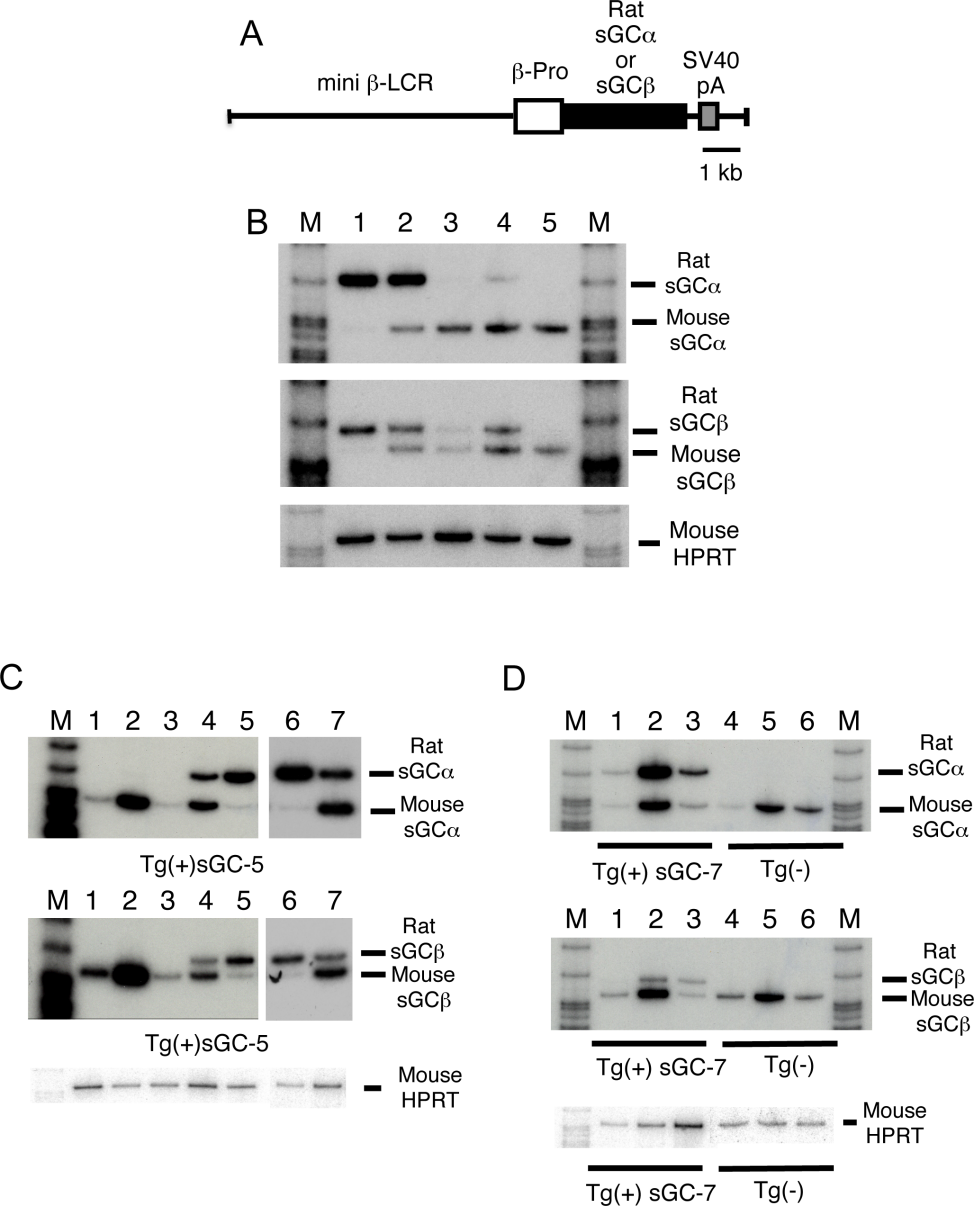
Generation and characterization of mice that overexpress rat sGC subunits in blood cells. **(A)** Structure of the transgenes used to generate sGC transgenic mice. Human β-globin gene promoter (β-pro) is ligated with a mini β-LCR (open box), cDNA encoding either rat sGCα or sGCβ (black box), and an SV40 intron poly(A) signal sequence (SV40pA) (gray box). Black bar is equivalent to 1 kilo-base (kb). **(B)** Transgene expression in sGC transgenic mouse lines. Expression was examined by reverse transcriptase-PCR using 1 μg total RNA prepared from BM cells. PCR products of rat sGCα (R-sGCα), rat sGCβ (R-sGCβ), mouse sGCα (M-sGCα), and mouse sGCβ (M-sGCβ) are shown in (B)-(D). Lanes: 1, sGC-5; 2, sGC-7; 3, sGC-8; 4, sGC-9; 5, non-transgenic (Tg(-)) mice; M, molecular weight marker. In (B)-(D), mouse hypoxanthine phosphoribosyltransferase (HPRT) was used as an internal control for equal loading. Note: Mouse endogenous sGC mRNAs were undetectable in sGC-5 mice (Lane 1) by RT-PCR. **(C)** Transgene expression in various tissues of sGC-5 mice. Reverse transcriptase-PCR was performed using 1 μg total RNA prepared from various tissues; heart (lane 1); lung (2); liver (3); spleen (4); BM cells (5); peripheral RBCs (6); and peripheral leukocytes (7); M, molecular weight marker. **(D)** Developmental stage-specific expression of transgenes in sGC-7 mice and non-transgenic littermates. Reverse transcriptase-PCR was performed using 1 μg total RNA of yolk sac at 10.5 dpc (lanes 1&4); fetal liver at 14.5 dpc (2&5); and adult BM cells (3&6).

To determine whether the NO-mediated hematologic effects seen in NO-treated mice involve sGC, we next generated mice which overexpressed rat sGC subunits in RBCs and leukocytes. We prepared plasmid constructs in which the sGC subunit cDNA was driven by the β-LCR and a β-globin gene promoter (Fig 2A) and injected two plasmid constructs into fertilized mouse eggs. Our construct was based on a transgene construct [34] which is expressed in both RBCs and the spleen, a myeloid/lymphoid tissue. We established four transgenic lines carrying both transgenes (sGC-5, 7, 8, &9) in B6CBA. The transgenes were expressed at high levels in sGC-5 and sGC-7, however endogenous mouse sGC mRNA was downregulated (Fig 2B lanes 1&2), a result consistent with the study by Filippov et al.[35]. A possible explanation is that mouse sGC mRNA degrades in cells with high cGMP levels as a consequence of activation of the sGC-cGMP pathway. Both transgenes were expressed in spleen lymphocytes (Fig 2C lane 4), BM cells (lane 5), peripheral blood RBCs (lane 6), and peripheral blood leukocytes (lane 7). During the development of erythroid cells, both transgenes were highly expressed in fetal livers at 14.5 days postcoitum (dpc) (Fig 1D lane 2) but at reduced levels in adult erythroid cells (lanes 3&5). Expression levels of rat and endogenous mouse sGC mRNAs were similar, suggesting that expression of the transgenes is regulated in a manner similar to that of endogenous mouse genes.

### Forced expression of sGC activates cGMP signaling in sGC mice

**Fig 3:**
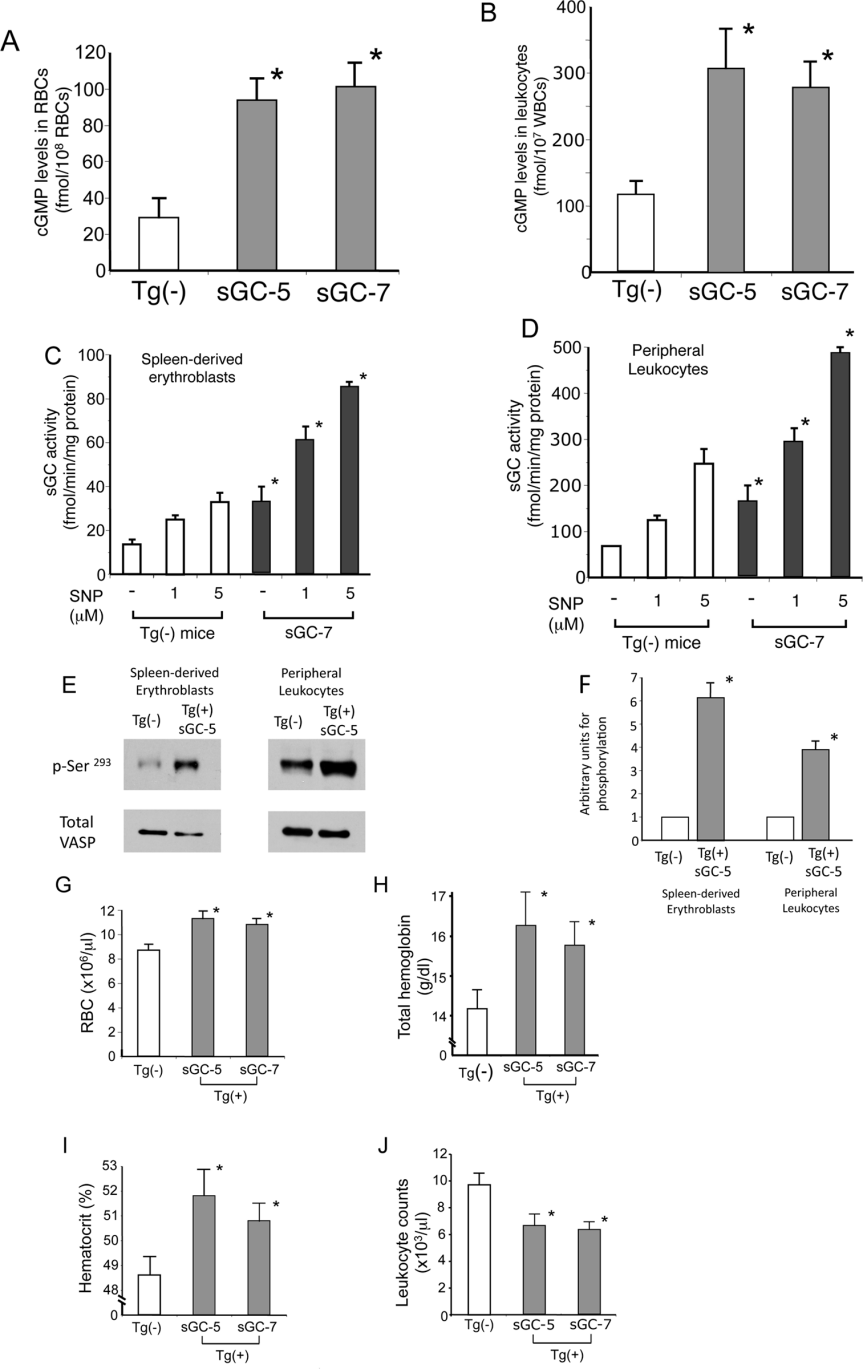
Examination of sGC activity in blood cells and hematologic analysis of sGC transgenic mice and non-transgenic (Tg(-)) littermates. **(A) (B)** Elevation of intracellular cGMP levels in sGC-5 mouse peripheral RBCs (A) and leukocytes (B). Intracellular cGMP levels of blood cells were measured using ELISA kits, as described in S1 Text. P values: *, *P*<0.01 compared with Tg(-) littermates. **(C) (D)** The sGC activity of spleen-derived erythroblasts and peripheral leukocytes are elevated in sGC mice. Cellular extracts prepared from erythroblasts or leukocytes were incubated with or without sodium nitroprusside (SNP; 0.1 or 5 μM) and sGC activity was determined by measuring the cGMP levels in the mixtures. P value: *, *P*<0.01 compared with Tg(-) littermates. **(E) (F)** Cellular extracts were subjected to immunoblotting to examine VASP phosphorylation in spleen-derived erythroblasts and peripheral leukocytes of sGC transgenic and Tg(-) mice. The experiment was repeated 3 times and a representative result is shown. Protein band intensity was quantified by the NIH image software Image J (version 1.63) and the results are summarized in Fig 2F. P value: *, *P*<0.01 compared with Tg(-) littermates. **(G-J)** Complete blood counts of sGC mice. RBCs (G), total hemoglobin (H), hematocrit (I), and leukocyte count (J) of sGC-5, sGC-7, and non-transgenic littermates were analyzed. See S2 Table for other hematologic values. P value: *, *P* < .05 compared with Tg(-) non-transgenic littermates.

We measured the intracellular cGMP level of RBCs and leukocytes to determine whether cGMP signaling is activated in sGC mice; we found them to be about 3 times higher than those of non-transgenic littermates (Fig 3A & 3B, P<0.01). Furthermore, the basal sGC activity of cytoplasmic preparations from spleen-derived erythroblasts and leukocytes was about 2 times higher than that of non-transgenic littermates (Fig 3C & 3D, P<0.01). Adding SNP (1 or 5 μM) to the cytoplasmic preparations increased sGC activity 2- to 3-fold (P<0.01). To further describe the status of cGMP signaling in sGC transgenic mouse erythroblasts and leukocytes, we examined phosphorylation of VASP, a substrate of cGMP-dependent protein kinase [36], and found it to be significantly higher in sGC transgenic mice than non-transgenic littermates (P<0.01) (see Fig 3E for results and 3F for summary). We found that cGMP signaling was activated in both spleen-derived erythroblasts and leukocytes of sGC mice.

To assess the effect of activated cGMP signaling, we compared the hematologic parameters of sGC mouse lines to non-transgenic littermates. Both sGC-5 and sGC-7 had elevated RBC counts, total hemoglobin, and hematocrit, but lower leukocyte counts (Fig 3G–3J) (P<0.05). There was no difference in the differential leukocyte count of sGC transgenic and non-transgenic littermates (data not shown). Other hematologic values are summarized in S2 Table. These results show that cGMP signaling exerts substantial hematologic effects in vivo.

### Effect of cGMP signaling on differentiation of hematopoietic progenitors

**Fig 4:**
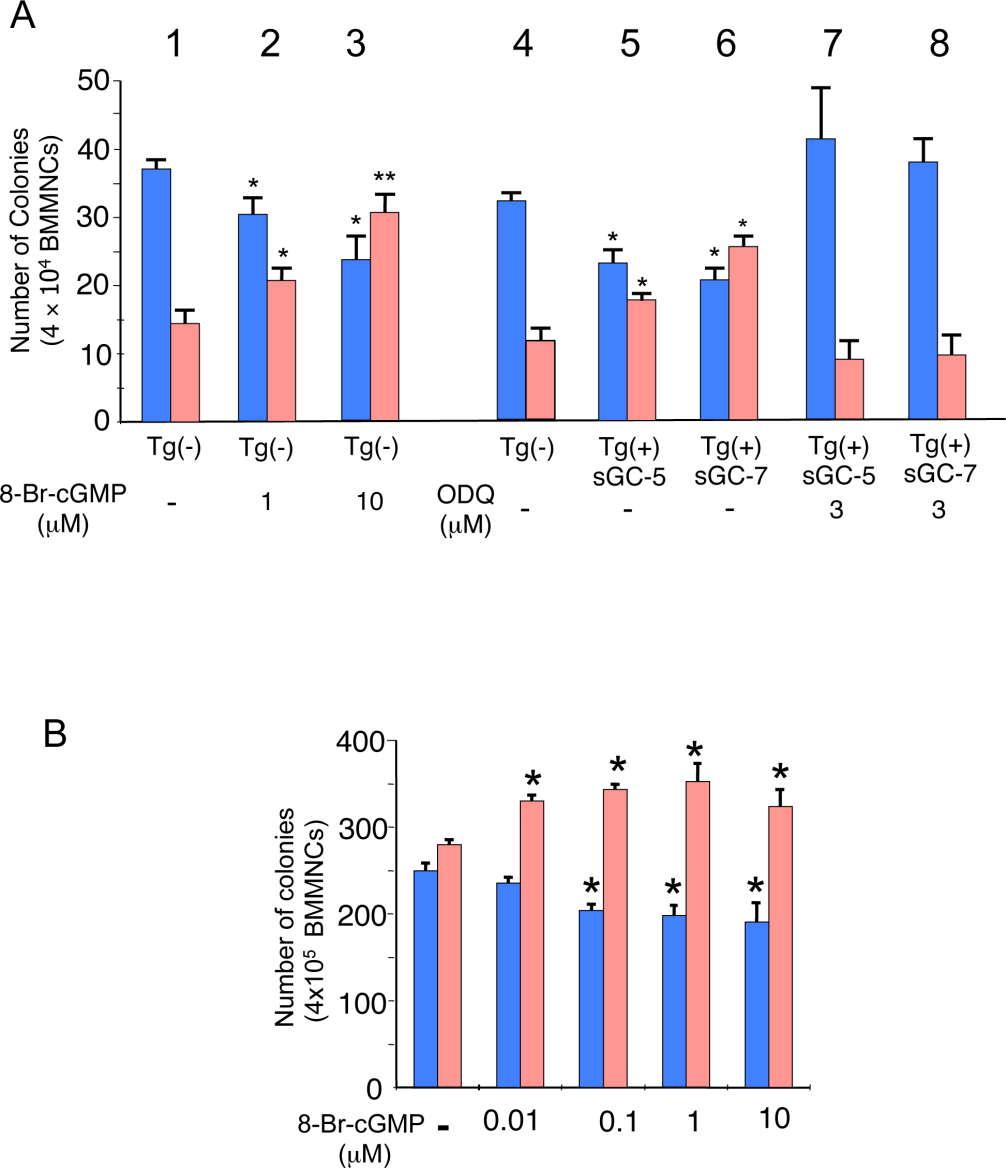
Effects of the sGC-cGMP pathway on cell differentiation of murine and human BM progenitors. **(A)** Effects of 8-bromo-cGMP and ODQ on the differentiation of murine BM progenitors prepared from sGC transgenic mice and Tg(-) littermates. BMMNC (4 × 10^4^ cells) were analyzed by semi-solid colony assays as described in S1 Text. Results are shown as total numbers of erythroid colonies (red column) and myeloid colonies (blue column). Origin of cells and treatment are as follows: lane 1, Tg(-) (control culture); lane 2, Tg(-) with 1 μM 8-bromo-cGMP; lane 3, Tg(-) with 10 μM 8-bromo-cGMP; lane 4, Tg(-); lane 5, sGC-5; lane 6, sGC-7; lane 7, sGC-5 with 3 μM ODQ; lane 8, sGC-7 with 3 μM ODQ. P values: *, *P*<0.05; **, *P*<0.01 compared with control culture. **(B)** Effects of 8-bromo-cGMP on the differentiation of human BM progenitors. Human BMMNC (4 × 10^5^ cells) were used for semi-solid colony assays as described in Materials and Methods and S1 Text. Results are shown as total number of erythroid colonies (red column) and myeloid colonies (blue column). Lanes: 1, no addition (control culture); 2, 0.01 μM 8-bromo-cGMP; 3, 0.1 μM 8-bromo-cGMP; 4, 1 μM 8-bromo-cGMP; 5, 10 μM 8-bromo-cGMP. P value: *, *P*<0.05 compared with control culture.

We investigated the cellular and molecular mechanisms underlying the changes to RBC and leukocyte levels in sGC mice. Semi-solid colony assays using BM cells prepared from non-transgenic littermates in the presence of various concentrations of 8-bromo-cGMP, a membrane-permeable cGMP analog [21] enhanced the differentiation of progenitors to erythroid-lineage cells but suppressed to myeloid-lineage cells (Fig 4A lanes 1 to 3) (P<0.05 to 0.01). In contrast, BM cells prepared from sGC mice demonstrated fewer myeloid but more erythroid colonies than those of non-transgenic littermates (Fig 4A lanes 4 to 6) (P<0.05). However, the sGC inhibitor ODQ abrogated such cGMP-mediated effects (Fig 4A lanes 7&8). Semi-solid colony assays showed that cGMP exerts the same effects on human hematopoietic progenitors (Fig 4B) (P<0.05) (see S1 Fig for the color of cell pellets treated with 8-bromo-cGMP).

**Fig 5:**
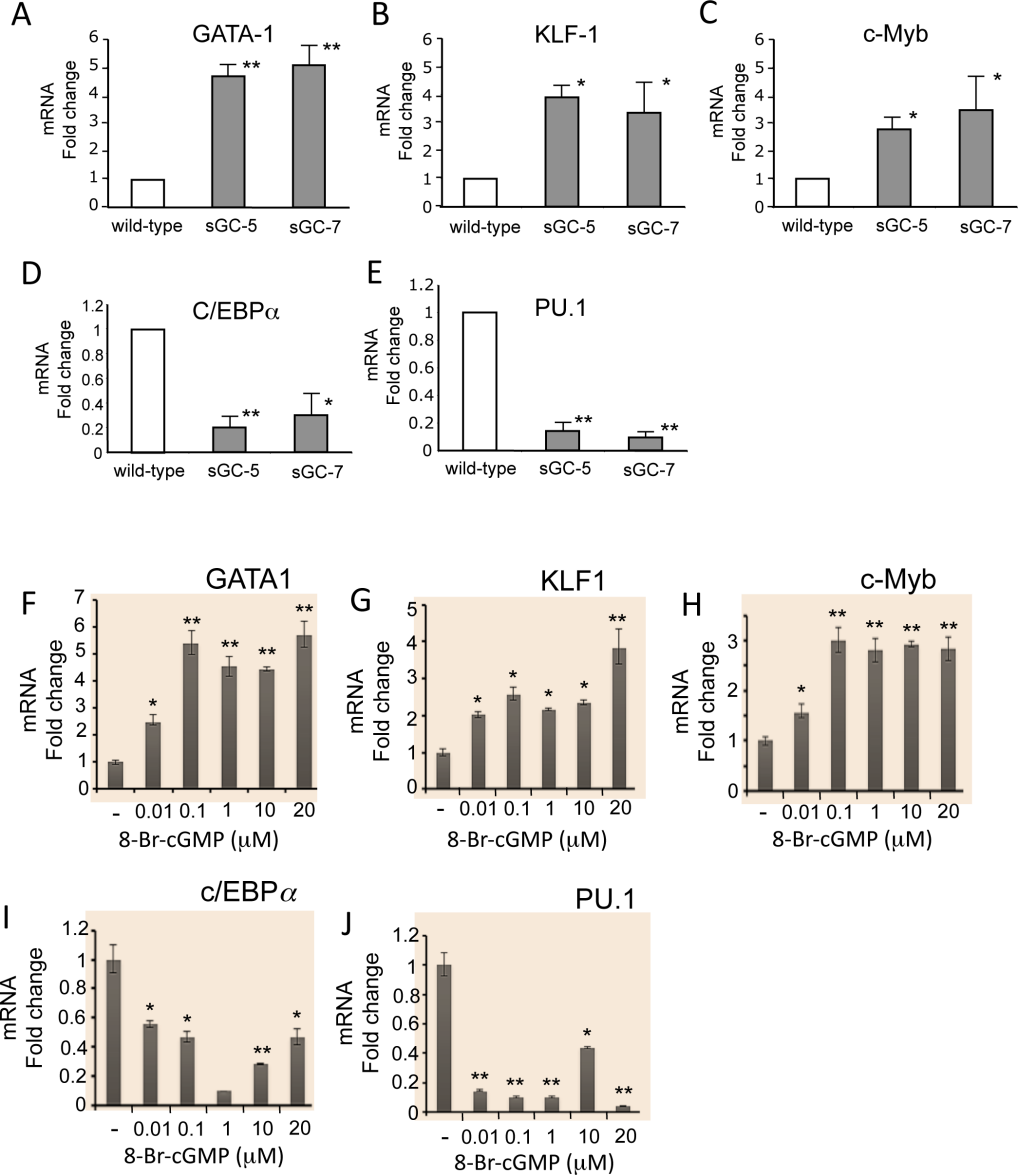
Effect of the sGC-cGMP pathway on expression of lineage cell-specific transcription factors in murine and human BM progenitors. **(A to E)** Altered expression of erythroid- and myeloid cell-specific transcription factors in sGC transgenic mice. Transcription factor expression was determined by RT-PCR as described in Materials & Methods using spleen-derived erythroid cells and peripheral blood leukocytes from sGC mice (sGC-5 and sGC-7, n = 4) and Tg(-) littermates (n = 5). Expression level of transcription factors in Tg(-) littermates (n = 4) was set to 1. P values: *, *P*<0.05; **, *P*<0.01 compared with Tg(-) littermates. **(F to J)** Effect of 8-bromo-cGMP on expression of erythroid- and myeloid cell-specific transcription factors in human BMMNC. Human BM cells (4 × 10^5^ cells) were mixed with Methocult in the presence or absence of various concentrations of 8-bromo-cGMP (0.01 to 20 μM). Cells were isolated from semi-solid colony plates and total RNAs were purified. Expression levels of transcription factors were determined by real time-PCR. P values: *, *P*<0.05; **, *P*<0.01 compared with control cultures.

We next examined the expression of erythroid- and myeloid-lineage transcription factors to determine whether the modulation by cGMP signaling of progenitor differentiation is associated with altered expression of lineage-specific transcription factors. We performed RT-PCR using spleen-derived erythroblasts and peripheral blood leukocytes isolated from sGC transgenic mice. In the sGC-5 and sGC-7 lines, the expression of transcription factors that play a role in erythroid cell differentiation, including GATA-1, KLF-1 and c-Myb, was elevated compared to those of non-transgenic littermates (Fig 5A–5C) (P<0.05 to P<0.01). However, the expression of myeloid cell-specific transcription factors such as c/EBPα and PU.1 was significantly lower than that of non-transgenic littermates (Fig 5D & 5E). Similarly, when human BM cells were treated with various concentrations of 8-bromo-cGMP, expression of GATA-1, KLF-1 and c-Myb was increased in a dose-dependent manner, but expression of myeloid cell-specific transcription factors such as c/EBPα and PU.1 was significantly suppressed (Fig 5F–5J)(P<0.05 to P<0.01). These effects are consistent with the colony assay results above (Fig 4). Thus, cGMP has been shown to have stimulatory and inhibitory effects on the differentiation of erythroid- and myeloid-lineage cells, respectively.

### Effect of cGMP signaling on human γ-globin expression

**Fig 6:**
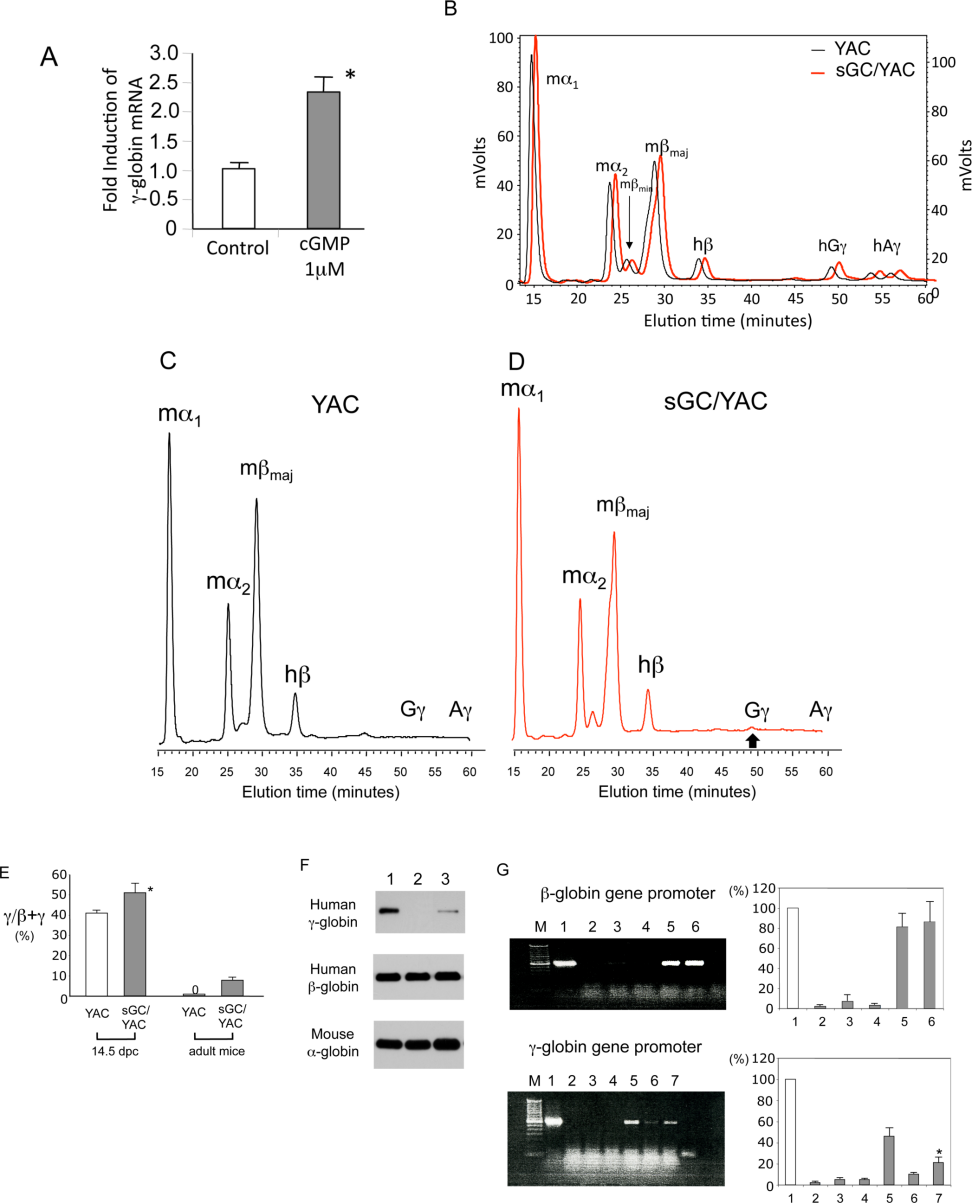
Examination of human β-like globin expression in sGC transgenic mice carrying a YAC clone (sGC-YAC) including the human β-globin gene locus. **(A)** 8-bromo-cGMP induces human γ-globin mRNA expression in human BM progenitors. P value: *, *P*<0.05 compared with control. **(B)** Expression of human γ-globin expression in the fetal liver (14.5 dpc) of YAC mice and sGC-YAC mice. Human β-globin was used as the internal control to compare the γ-globin expression levels of YAC and sGC-YAC mice. Globin chain analysis in fetal livers was performed by reverse-phase HPLC as described in S1 Text. Representative elution profiles are shown from 3 independent experiments that gave similar results. **(C) (D)** Expression of human γ-globin expression in adult YAC mice (C) and sGC-YAC mice (D). Globin chains in peripheral blood prepared from YAC or sGC-YAC mice were analyzed by reverse-phase HPLC. In (D) upward bold arrow indicates the position of Gγ-globin in sGC-YAC mice. Note: Gγ-globin, but not Aγ-globin, was detected in RBCs from adult sGC-YAC mice. **(E)** Summary of human β-like globin expression in YAC mice and sGC-YAC mice during development. P value: *, *P*<0.05 compared with YAC mice. **(F)** Detection of human γ-globin in mouse RBCs. Total cell lysates (10 μg) prepared from mouse RBCs were analyzed in 12% SDS-PAGE gels by immunoblotting as described [31] Lanes: 1, RBCs from fetal livers of YAC mice (14.5 dpc); 2, peripheral blood RBCs from adult YAC mice; 3, peripheral blood RBCs from adult sGC-YAC mice. **(G)** Recruitment of RNA polymerase II to β-like globin gene promoters in sGC-YAC mice. ChIP assays were performed using spleen-derived erythroblasts as described in S1 Text. Upper panel: 1, input DNA; 2, non-specific IgG antibody; 3, brain; 4, sGC mice; 5, sGC-YAC mice; 6, YAC mice. Lower panel: 1, input DNA; 2, non-specific IgG antibody; 3, brain; 4, sGC mice; 5, fetal liver cells (14.5 dpc) of sGC-YAC mice; 6, YAC mice;7, adult erythroid cells from sGC-YAC mouse. Representative results are shown from experiments that were repeated at least 3 times. *, *P*<0.05.

We previously demonstrated that human γ-globin gene expression is induced by activating the sGC-cGMP pathway in primary erythroid cells [21]. In this study, we found that 1 μM 8-bromo-cGMP consistently enhanced γ-globin mRNA expression levels by 2.5-fold in human BM cells (Fig 6A) (*P*<0.05). To examine the effect of cGMP signaling on γ-globin expression in vivo, we bred sGC transgenic mice with a YAC mouse carrying the entire human β-globin gene locus [27]. In sGC-YAC mice (n = 5), the ratio of γ-globin to the sum of β- and γ-globin in fetal livers (14.5 dpc) increased by approximately 10% compared to YAC mice (Fig 6B). Although human γ-globin was not detected in adult YAC mice (Fig 6C), a low-level expression of human Gγ-globin, but not Aγ-globin, which is 8.3 ± 0.7% of the total output of β- and γ-globin, was observed in sGC transgenic mice carrying the YAC clone (Fig 6D). YAC and sGC-YAC mouse γ-globin expression levels are summarized in Fig 6E. We confirmed the expression of human γ-globin in the adult RBCs of sGC-YAC mice by immunoblotting (Fig 6F). To investigate the molecular mechanisms by which human γ-globin expression is induced in adult sGC/YAC mice, we performed ChIP assays and examined the interactions between the β- and γ-globin gene promoters and RNA polymerase II (Fig 6G). While enrichment of the β-globin gene promoter by RNA polymerase II antibody of sGC-YAC and YAC mice was comparable (upper panel lanes 5&6), the recruitment of RNA polymerase II to the Gγ-globin gene promoter was significantly higher in erythroblasts prepared from sGC-YAC mice than in those from YAC mice (lower panel, lanes 6&7) (*P*<0.05). These results indicate that cGMP signaling modulates HbF expression at the level of transcription, and are consistent with our previous report that the sGC-cGMP pathway is involved in γ-globin gene expression [21].

## Discussion

Although there are numerous studies of the physiologic effects of NO-cGMP signaling [37] [38], little is known about its hematologic effects in vivo. Because sGC knock-out mice show a lethal phenotype [19] and the cGMP levels are elevated in RBCs of SCD patients [24], we examined the hematological influences of activated NO-cGMP signaling in vivo. An important finding of this study is that both NO-treated mice and sGC transgenic mice, in which cGMP signaling was activated in multiple cell types, demonstrate increases in total hemoglobin and hematocrit but a reduction in leukocyte count (Figs 1 to 3). This suggests that, when activated, NO-cGMP signaling exerts positive regulatory activity on erythropoiesis but inhibits myelopoiesis. This notion is supported by our in vitro studies, in which a cGMP analog stimulated the differentiation of hematopoietic progenitors to erythroid-lineage cells but inhibited them from differentiating to myeloid-lineage cells (Fig 1F to K, Fig 4). Such regulatory effects of NO-cGMP signaling on cell differentiation are likely supported by simultaneously controlling the expression of lineage-specific transcription factors including GATA-1, KLF-1, cEBP/α, and PU.1 (Fig 5). These results demonstrate the molecular basis for the hematologic changes seen in NO-treated and sGC transgenic mice. Consistent with our previous study [21], γ-globin expression in sGC-YAC mice [27] was upregulated at a low but appreciable level, as confirmed by HPLC and immunoblotting. Our ChIP assays verified the increased interactions between RNA polymerase II and the γ-globin promoter in erythroblasts isolated from sGC-YAC mice (Fig 6G). Taken together, our results show that NO-cGMP signaling modulates hematopoiesis by simultaneously regulating the expression of multiple lineage-specific transcription factors and plays a role in γ-globin expression in vivo. cGMP, though ubiquitously distributed among multiple types of cells, is likely to exert lineage-specific hematologic activities.

A recent study reported that oral administration of sodium nitrate suppresses erythropoiesis in rats [39], which appears to contradict our findings. However, inasmuch as plasma nitrate levels were elevated in our NO-treated mice (data not shown), elevated levels of plasma NO metabolites could constitute a negative feedback mechanism for erythropoiesis. An alternative explanation could be that there might be substantial differences in the mechanisms regulating erythropoiesis between rats and mice; for instance, the half life of RBCs in rats is shorter than that of mice [40,41].

Our results have clinically relevant implications for anemic disorders such as SCD. First, hematologic changes such as increased total hemoglobin and HbF expression as well as reduced leukocyte count occur among SCD patients treated with HU [42]. Because intracellular cGMP increases in RBCs in response to HU therapy [43], it is possible that HU activates cGMP signaling through NO production [17,23,44]. There is a striking resemblance between the hematologic changes seen in mice in whom NO-cGMP signaling was activated, either by NO inhalation or sGC overexpression, and the hematologic response of SCD patients undergoing HU therapy (Figs 1–3). Thus, it appears that NO-cGMP signaling may be involved in part in the HU-induced hematologic changes seen in SCD patients. Second, and more importantly, because activation of cGMP signaling stimulates erythropoiesis (Fig 3), chemicals capable of activating cGMP signaling [6,45] could potentially be developed into treatments for anemia. This is particularly important in light of their oral availability, while erythropoietin, the only cytokine that is able to stimulate erythropoiesis [46], must be injected intravenously. Collectively, these findings suggest that NO-cGMP signaling exerts broader hematologic effects than HbF expression [21].

Our finding that NO-cGMP signaling plays a role in modulating erythropoiesis as well as γ-globin gene expression may account for some puzzling clinical and experimental observations. First, the negative correlation found between HbF and zinc protoporphyrin levels in SCD [47] may be explained by the fact that zinc protoporphyrin is a strong sGC inhibitor [48] and could reduce HbF expression in this disorder. Second, Lutton et al. reported that zinc protoporphyrin inhibits hematopoiesis [49], which is a conclusion compatible with our finding that the sGC-cGMP pathway is important to hematopoiesis (Figs 3–5). Third, in contrast to other porphyrias, anemia is uncommon in patients with erythropoietic protoporphyria [50], but the underlying mechanism remains unclear. Our current studies suggest that protoporphyrin IX, an sGC activator [51] which is elevated in this disorder, may stimulate erythropoiesis and supplement hemoglobin levels through sGC-cGMP signaling. Fourth, previous studies reported an inverse correlation between hemoglobin and NO metabolite levels in anemic conditions [52,53], suggesting that NO is a causative factor for anemia. Our study, however, suggests that activation of NO-cGMP signaling in these disorders may in fact constitute a hematologic adaptive response to correct anemic conditions, as was recently suggested [54]. These multiple lines of prior evidence support the important role of NO-sGC-cGMP signaling in the regulation of hematopoiesis including globin gene expression.

Several lines of clinical evidence suggest that the mechanisms regulating leukocyte count may be involved in HU-mediated HbF induction in the context of SCD. First, robust HbF induction by HU is associated with a pronounced reduction in leukocyte and reticulocyte counts [55]. We recently showed that HU-mediated HbF induction in SCD patients is correlated with HU-associated leukocyte reduction [56]. Furthermore, in SCD patients with hereditary persistence of HbF, HbF levels were inversely correlated with leukocyte count [56]. Interestingly, leukocyte count tends to increase in SCD patients who become resistant to HU treatment [42].

**Fig 7:**
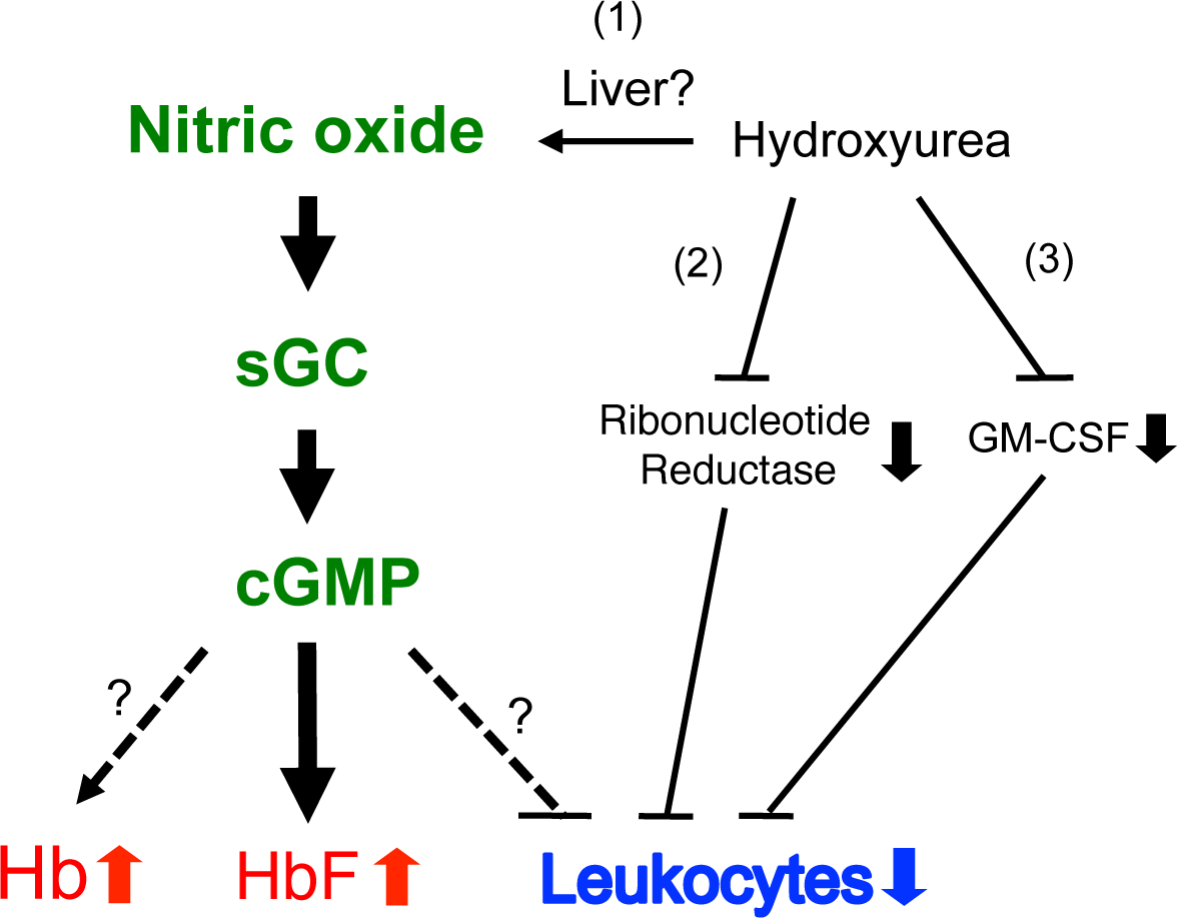
Model of the molecular actions of NO-cGMP signaling and HU. While the current study has shown hematologic effects of NO-cGMP signaling on RBCs and leukocytes, the molecular actions of HU are yet to be elucidated. NO may be generated when HU is metabolized in the liver (pathway 1). HU may reduce leukocyte counts by inhibiting DNA synthesis through ribonucleotide reductase (pathway 2) [58] or GM-CSF (pathway 3) [56]. cGMP induces HbF expression [21] and inhibition of ribonucleotide reductase may account in part for leukocyte reduction and HbF induction [61]. This study suggests that cGMP may also be associated with a rise in total hemoglobin (Hb) and a decrease in leukocyte count. Solid lines represent molecular actions noted in the literature and dotted lines indicate those examined in this study.

The HU-mediated reduction in leukocyte count may occur via one or more pathways (Fig 7): the generation of NO which induces apoptosis in leukocytes (pathway 1) [57]; the inhibition of DNA synthesis through the suppression of ribonucleotide reductase (pathway 2) [58]; or mediation by a reduction in granulocyte macrophage colony-stimulating factor (GM-CSF), which has a role in leukocytosis in SCD (pathway 3) [43]. Our current study introduces a novel mechanism for leukocyte reduction: NO-cGMP signaling prevents progenitor differentiation to myeloid-lineage cells by regulating lineage-specific transcription factors (Figs 4 & 5). HU increases intracellular cGMP levels, presumably through NO generation [23,24], and cGMP delays the G to S phase transition by inhibiting cyclin D1 expression and cyclin-dependent kinase 4 activation [59], which may contribute to leukocyte reduction by HU.

## Conclusions

In conclusion, this study shows for the first time that activation of NO-cGMP signaling in blood cells induces erythropoiesis, which is associated with increases in RBC counts and total hemoglobin levels, but suppresses myelopoiesis by reducing leukocyte counts. Altered expression of lineage-specific transcription factors caused by cGMP signaling may contribute to these hematopoietic changes in NO-treated mice and sGC transgenic mice. Our finding that peripheral blood leukocyte counts can be reduced by activating cGMP signaling suggests that leukocyte count could serve as a good surrogate marker for evaluating the sGC-cGMP pathway in SCD patients who receive a phosphodiesterase 5 inhibitor or other sGC-cGMP pathway activator [45]. Administration of cGMP-elevating agents together with HU may have immediate clinical effects in patients with SCD [60], suggesting a new combination therapy. Further studies of the mechanisms underlying hematologic changes in SCD patients receiving HU may provide insight about HU resistance. More importantly, in addition to erythropoietin, the NO-cGMP signaling axis may constitute a novel signaling target pathway to regulate erythropoiesis in vivo.

## Supporting Information

S1 Fig Effects of membrane-permeable cGMP on the differentiation of human bone marrow progenitors. (PDF)

S1 Table Copy number of the transgenes in sGC transgenic mice. (PDF)

S2 Table Hematologic data of sGC transgenic mice and non-transgenic littermates. (PDF)

S1 Text Detailed Procedures for Materials & Methods. (PDF)

## Abbreviations

ChIP: chromatin immunoprecipitation
HbF: fetal hemoglobin
HU: hydroxyurea
HPLC: high-performance liquid chromatography
NO: nitric oxide
RBCs: red blood cells
SCD: sickle cell disease
sGC: soluble guanylate cyclase
SNP: sodium nitroprusside
Tg(+): transgenic
Tg(-): non-transgenic
YAC: yeast artificial chromosome
