# Supplementary material for: Nitric Oxide-cGMP Signaling Stimulates Erythropoiesis through Multiple Lineage-Specific Transcription Factors: Clinical Implications and a Novel Target for Erythropoiesis
Source: PLoS One. 2016 Jan 4;11(1):e0144561. doi: 10.1371/journal.pone.0144561 (PMC4699757; doi:10.1371/journal.pone.0144561)
Supplement: S1 Fig — (PDF) [file pone.0144561.s001.pdf]

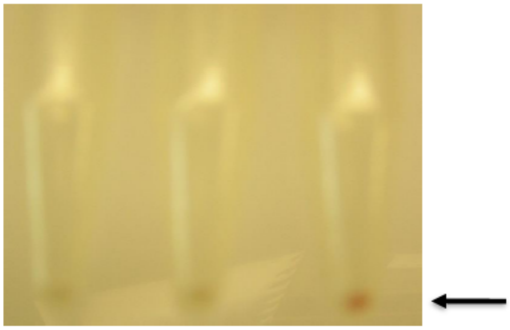

Fig. S1: Effects of membrane-permeable cGMP on the differentiation of human bone marrow progenitors.

Materials and Methods:

Human BM cells (4 x10^5) were mixed with 4 ml of MethoCult (Cat.# GF H4434, Stem Cell Techonolgies). Following the addition of 8-Br-cGMP shown above, the cells were dispensed into 35-mm dishes. Semi-solid cultures were kept for 14 days in a humidified CO_2 incubator at 37 °C. Colony forming cells were then counted under a phase contrast inversion microscope. Arrow indicate the cell pellets. Note that the cell pellet treated with 1 μM 8-Br-cGMP is reddish, suggesting amplification of erythroid-lineage cells.
