# Supplementary material for: Nitric Oxide-cGMP Signaling Stimulates Erythropoiesis through Multiple Lineage-Specific Transcription Factors: Clinical Implications and a Novel Target for Erythropoiesis
Source: PLoS One. 2016 Jan 4;11(1):e0144561. doi: 10.1371/journal.pone.0144561 (PMC4699757; doi:10.1371/journal.pone.0144561)
Supplement: S1 Table — (PDF) [file pone.0144561.s002.pdf]

Table S1. Copy number of the transgenes in sGC transgenic mice *

Line #	sGCα (copies)	sGCβ (copies)
sGC-5	4	4
sGC-7	3	2
sGC-8	1	2
sGC-9	3	3

* Copy numbers of sGC transgenic mice were determined as described above.
