# Supplementary material for: Nitric Oxide-cGMP Signaling Stimulates Erythropoiesis through Multiple Lineage-Specific Transcription Factors: Clinical Implications and a Novel Target for Erythropoiesis
Source: PLoS One. 2016 Jan 4;11(1):e0144561. doi: 10.1371/journal.pone.0144561 (PMC4699757; doi:10.1371/journal.pone.0144561)
Supplement: S2 Table — (PDF) [file pone.0144561.s003.pdf]

Table S2 Hematologic data of sGC transgenic mice and non-transgenic littermates.

	Tg(-) mice (n=7)	sGC-5 (n=6)	sGC-7 (n=5)
RBC ($10^6/\mu l$)	8.7 ± 0.4	11.3 ± 0.6 *	10.8 ± 0.4 *
Hemoglobin (g/dl)	14.2 ± 0.6	16.3 ± 1.1 *	15.8 ± 0.8 *
Hematocrit (%)	48.6 ± 0.8	51.8 ± 1.4 *	50.8 ± 0.9 *
MCV (fl)	50.7 ± 2.1	46.7 ± 3.3	47.0 ± 3.7
MCH (pg/cell)	14.8 ± 0.8	14.5 ± 0.9	14.6 ± 1.0
MCHC (g/dl)	29.8 ± 1.4	31.4 ± 1.2	31.1 ± 1.1
Reticulocytes (%)	3.9 ± 0.5	3.0 ± 0.7	3.3 ± 0.6
Leucocytes ($10^3/\mu l$)	9.8 ± 1.1	6.7 ± 1.5 *	6.4 ± 0.8 *
Platelets ($10^6/\mu l$)	1.09 ± 0.18	1.12 ± 0.33	1.04 ± 0.21

Hematologic data of each mouse group were obtained from 5 to 7 mice.

* $P < 0.05$ compared to Tg(-) non-transgenic littermates.
