# Supplementary material for: Nitric Oxide-cGMP Signaling Stimulates Erythropoiesis through Multiple Lineage-Specific Transcription Factors: Clinical Implications and a Novel Target for Erythropoiesis
Source: PLoS One. 2016 Jan 4;11(1):e0144561. doi: 10.1371/journal.pone.0144561 (PMC4699757; doi:10.1371/journal.pone.0144561)
Supplement: S1 Text — (PDF) [file pone.0144561.s004.pdf]

Nitric oxide-cGMP signaling stimulates erythropoiesis through multiple lineage-specific transcription factors in vitro and in vivo: A novel target for erythropoiesis

Tohru Ikuta,[1] Hassan Sellak,[1] Nadine Odo,[1] Adekunle D. Adekile,[2] and Karin M. L. Gaensler [3]

[1] Department of Anesthesiology and Perioperative Medicine, Medical College of Georgia, Georgia Regents University, Augusta, Georgia, U.S.A., [2] Department of Paediatrics, Faculty of Medicine, Kuwait University, Safat, Kuwait, and [3] Division of Hematology/Oncology, Department of Medicine, University of California, San Francisco, California, U.S.A.

Materials and Methods

Preparation of sGC constructs

A 2.1-kb DNA fragment containing rat sGCα cDNA or a 1.9-kb DNA fragment containing rat sGCβ cDNA [1,2] was linked to NotI/XbaI site of pcDNA 1/Amp (Invitrogen, Carlsbad, CA, USA). A 0.9-kb DNA fragment containing a human β-globin gene promoter (provided by J. C. Chang, University of California San Francisco) was amplified using Vent DNA polymerase (New England Biolabs, Beverly, MA) with primers carrying Hind III and Not I sites, and placed upstream to rat sGCα or sGCβ cDNA. Next, a 6.5-kb fragment containing a human β-globin locus control region (β-LCR) was released from pSPT/βLCR/a-pro/α-glo [3] (provided by J. C. Chang, University of California San Francisco). To add β-LCR 5' to the β-globin gene promoter, a 3.7-kb DNA fragment containing β-promoter/sGCα-SV40 intron/polyA (SV40 intron/polyA is located downstream of the multiple cloning site of pcDNA 1/Amp) was amplified using primers with Cla I and Sal I, and linked with pBCβ-LCR that was created by ligating a 6.5-kb DNA fragment containing β-LCR to pBC SK (-) (Agilent Technologies, Santa Clara, CA, USA) using Sac II. Similarly, a 3.5-kb DNA fragment containing β-promoter-sGCβ-SV40 intron/polyA was amplified using primers with EcoR I or Sal I, and inserted 3'' to pBCβ-LCR. The plasmid constructs were sequenced by the dideoxy termination method [4] using the ABI 3730 XL 96-capillary sequencer (Applied Biosystems, Foster City, CA, USA) and Big Dye Terminator 3.1 (Applied Biosystems).

Generation of sGC transgenic mice

A 10.4-kb DNA fragment containing a βLCR-βpro-sGCα-SV40 intron/polyA and a 10.1-kb DNA fragment containing βLCR-βpro-sGCβ-SV40 intron/polyA were released from the constructs by digestion with BssH II or EcoR V/Sal I and purified by agarose gel electrophoresis and a QIAEX II gel extraction kit (Qiagen, Valencia, CA, USA). Both DNA fragments were dissolved in 10 mM Tris-Cl/0.1 mM EDTA (pH 7.4) and coinjected into fertilized B6CBA eggs. The eggs were transferred to pseudopregnant B6CBA foster mothers and allowed to develop to term. Founder mice carrying both sGCα and sGCβ were identified by PCR using tail DNA with the following three primers: forward β-pro-F3 (common to sGCα and sGCβ), 5'-AAAACGATCTTCAATATGCT-3'; reverse sGCα, 5'-GAGCTTGCCAATACTCTCTG-3'; reverse sGCβ, 5'-ATAATCCCGATCACAATGTC-3'. Founder mice were bred with non-transgenic B6CBA mice to generate F1 progeny. If necessary, mice were anesthetized with ketamine/xylazine (0.1mg/0.015mg/g, IP) and euthanasia was performed by cervical dislocation with anesthesia stated above. This study has been approved by the Institutional Animal Care and Use Committees of the Georgia Regents University and the University of California San Francisco.

Determination of copy numbers of sGC transgenes in sGC transgenic mice

To determine the copy numbers of sGC transgenes, we performed Southern blot and real-time quantitative PCR using genomic DNA isolated from the tails of 3-4 week-old sGC transgenic mice. Southern blotting was carried out by isolating 10 μg of genomic DNA which was digested with Nco I, fractionated in 7% agarose gels, and transferred to Zeta-Probe blotting membranes (Bio-Rad Laboratories, Hercules, CA, USA). The filters were hybridized with ^{32}P-labeled probes for the human β-globin promoter, SV40 intron/polyA, and mouse cAMP response element-binding protein (CREB) in ULTRAhyb hybridization buffer (Ambion, Austin, TX, USA) at 42 °C overnight. The probes were labeled with γ-^{32}P dCTP using random priming [5] (NEBlot Kit, New England BioLabs, Beverly, MA, USA). After washing, the filter was exposed to an x-ray film. A mouse CREB probe was used as an internal control to monitor equal loading.

Real-time semi-quantitative PCR was performed as follows: Genomic DNA (10 ng) was added to a 25-µl reaction volume containing 1x Brilliant SYBR Green QPCR Master Mix, 30 nM reference dye (Agilent Technologies), and 200 nM (each) of a primer set for a sGCα- or sGCβ-subunit. Primers for sGCα and sGCβ were designed so that rat sGC cDNA sequences and endogenous mouse sGC mRNA sequences were amplified simultaneously. The primers used were:

c-sGCα-F5, 5'-AGAAGGCTGGTGAACAAGAG-3';

c-sGCα-R5, 5'-AGTTATCCCATCTCCTCACC-3';

c-sGCβ-F5, 5'-TGCCAATGAGCTGAGACACA-3';

c-sGCβ-R5, 5'-GACAATCTTCATGGCCCCTT-3'.

Real-time PCR amplification and signal detection were performed using Mx3000P (Agilent Technologies) with the following conditions: initial denaturing at 95°C for 10 minutes followed by 40 cycles of 95 °C for 30 seconds, 60 °C for 1 minute, and 72 °C for 1 minute. The copy number was calculated using standard curves generated with a serial dilution of wild-type mouse genomic DNA.

Expression of rat sGC mRNAs in sGC transgenic mice

Expression of rat sGC mRNA in sGC transgenic mice was examined by reverse transcriptase PCR rather than real-time PCR to visually quantify expression levels of transgenes by autoradiography. Total RNA was prepared from the bone marrow (BM), spleen, heart, lung, liver, and peripheral blood leucocytes of sGC transgenic mice and non-transgenic mice by the method of Chomczynski and Sacchi [6]. RNA preparations were treated with RQ1 RNase-free DNase (Promega, Madison, WI, USA) at 37°C for 30 minutes to remove contaminated genomic DNA. First strand synthesis was carried out using 1 µg total RNA and the First-Strand cDNA Synthesis Kit (Amersham Biosciences, Piscataway, NJ, USA). The PCR reaction was performed with Platinum Taq DNA polymerase (Invitrogen, Carlsbad, CA, USA) and primers for sGCα or sGCβ that amplified both rat and mouse sGC sequences simultaneously. The mouse hypoxanthine phosphoribosyltransferase (HPRT) gene was used as an internal control. Primer sequences used in this study were as follows:

c-sGCα-F4, 5'-AGTGTGGAGAGCTGGATGT-3';

c-sGCα-R3, 5'-GGGCTGACATTGATTTTCCG-3';
c-sGCβ-F4, 5'-AAGGGGCCATGAAGATTGTC-3';
c-sGCβ-R3, 5'-GCATCCGCTGTCCAATCA-3';
mouse HPRT-F, 5'-GCTGGTGAAAAGGACCTCT-3';
mouse HPRT-R, 5'-CACAGGACTAGAACACCTGC-3'.

One μl of α-^{32}P dCTP (3000 Ci/mMole, Perkinelmer, Waltham, MA, USA) was added to radiolabel PCR products. PCR reactions included initial denaturing at 94 °C for 5 minutes, denaturing at 94 °C for 30 seconds, annealing at 60 °C for 30 seconds, and extension at 72 °C for 30 seconds. PCR was repeated 28 cycles except for the PCR with adult peripheral leucocyte cDNA, which was repeated for 30 cycles. PCR products from mouse sGCα and sGCβ were distinguished from those of rat sGCα and sGCβ by Nci I digestion. PCR products that were digested with Nci I were separated with 8% polyacrylamide gels and exposed to an x-ray film. Radioactivity of the individual bands was quantified by PhosphorImager Storm 820 (Amersham Biosciences, Piscataway, NJ, USA).

Intracellular cGMP levels in peripheral blood cells of sGC transgenic mice

Peripheral blood was collected from tails of anesthetized mice into EDTA-coated blood collection tubes. Immediately, 3-isobutyl-1-methylxanthine (IBMX) was added to blood at the final concentration of 1 mM and the mixture was incubated for 30 minutes at room temperature. Peripheral RBCs and leucocytes were isolated by density gradient centrifugation using Histopaque 1083 (Sigma Chemicals, St. Louis, MO, USA). Cyclic nucleotides were extracted by suspending the cells with 0.5 M perchloric acid. The supernatant was removed and the pH was neutralized by the addition of 8 M KOH. Intracellular cGMP levels were determined by an acetylation method (cGMP ELISA kit, Cayman Laboratories, Ann Arbor, MI, USA).

Measurement of sGC activities in mouse blood cells

Erythroblast-rich cells were isolated from the spleen following 3-day intraperitoneal injections of phenylhydrazine (0.4 mg for 10 g of mouse weight) into adult mice [7]. Peripheral leucocytes were isolated by density gradient centrifugation

with Histopaque 1083 (Sigma Chemicals). Cells were sonicated in 50-100 µl of lysis buffer (25 mM Tris-Cl, pH 7.4, 137 mM NaCl, 2.7 mM KCl, 4 mM $MgCl_2$, 0.1 mM PMSF, 2.5 µg/ml leupeptin, 2.5 µg/ml aprotinin, and 0.5 mM IBMX), and cellular extracts were prepared by centrifugation at 14,000 × g for 30 minutes. Thirty micrograms of the cellular extracts were incubated in 100 µl of assay buffer (25 mM Tris-Cl, pH 7.4, 137 mM NaCl, 2.7 mM KCl, 4 mM $MgCl_2$, 100 µM GTP, 5 mM phosphocreatine, 5 U/ml creatine phosphokinase, and 0.5 mM IBMX) at 37 °C for 15 minutes in the presence or absence of sodium nitroprusside (SNP; 0.1 or 5 µM). Reactions were terminated by adding 12 µl of 50% trichloroacetic acid. cGMP was extracted with diethyl ether three times and quantified using the cGMP enzyme immunoassay system (Amersham Biosciences, Piscataway, NJ, USA), according to the supplier's protocol. Experiments were repeated three times; representative results are shown in Fig.3C&D.

Examination of VASP phosphorylation by immunoblotting

Whole cellular extracts were prepared from spleen-derived erythroblasts and peripheral leucocytes of sGC mice and non-transgenic mice, as described previously.[8] Briefly, cells (5 to 10 × 10^6 cells) were suspended with 1 × lysis buffer (Santa Cruz Biotechnology, Santa Cruz, CA) supplemented with phosphatase inhibitor cocktail set IV (2% vol/vol) (Calbiochem, San Diego, CA). Whole cellular extracts were obtained by spinning at 14,000g for 15 minutes at 4 °C. Western blotting was performed as described previously.[9] Twenty to 30 µg of the cellular extracts were separated on 12 % SDS polyacrylamide gels and transferred to nitrocellulose membranes (Invitrogen, Carlsbad, CA, USA). VASP antibodies were purchased from Cell Signaling Technology unless otherwise stated. Protein bands were visualized by the Phototope HRP western blot detection system (Cell Signaling Technology), according to the protocol provided by the supplier.

Haematological analysis of sGC mice and non-transgenic littermates

Peripheral blood was collected from tails of anesthetized mice into EDTA-coated blood collection tubes. RBC count, haemoglobin concentration, haematocrit, mean corpuscular volume, mean corpuscular haemoglobin content, and mean corpuscular

haemoglobin concentration were measured using an automatic blood cell analyzer (Coulter A^c•T diff analyzer, Beckman Coulter, Fullerton, CA, USA). Reticulocyte counts were examined manually using blood smears stained with new methylene blue.

Semi-solid colony assays using murine and human BM progenitors

The membrane permeable cGMP analog 8-Bromo-cGMP and ODQ were purchased from Enzo Life Sciences (Farmingdale, NY, USA). To evaluate the effects of the sGC-cGMP pathway on the differentiation of hematopoietic progenitors, semi-solid cultures were performed using murine BM cells from sGC transgenic mice (sGC-5 and sGC-7) or human BM cells [8]. Briefly, murine BM cells (4×10^4 cells) or human BM cells (4×10^5 cells, Poietics, Walkersville, MD, USA) were mixed with 4 ml of MethoCult (GF M-3434 for murine BM cells, GF H4034 for human BM cells, Stem Cell Technologies, Vancouver, Canada). Following the addition of appropriate concentrations of 8-Bromo-cGMP or ODQ (3 μM), the cells were dispensed into 35-mm dishes. Semi-solid cultures were kept for 14 days in a humidified CO_2 incubator at 37°C. Colony forming cells were then counted under a phase contrast inversion microscope. Experiments were repeated three times using cells obtained from three independent sGC transgenic mice, non-transgenic littermates, and normal human subjects. BFU-Es and CFU-Es were classified as erythroid colonies and CFU-Gs and CFU-GMs as myeloid colonies [8].

Expression of lineage-specific transcription factors in murine and human BM progenitors treated with 8-Bromo-cGMP

Expression of lineage-specific transcription factors in spleen-derived erythroid cells and peripheral blood leukocytes prepared from sGC mice was examined by real time (RT)-PCR as described below. To determine the expression level of human transcription factors in erythroid and myeloid cells, human $CD34^+$ cells (4×10^5 cells) were cultured in the presence of 8-Bromo-cGMP as described [10]. Total RNA was extracted from individually plucked colonies using RNeasy Mini Kit (Qiagen). cDNA was generated with the SuperScript II Reverse Transcriptase kit (Invitrogen). RT PCR was carried out with the Mx3000p System (StrataGen) using iQ SYBR Green Super mix (Bio-Rad) according to the manufacturer’s instructions. All amplifications were

performed in triplicate, and 18S rRNA was used as the internal control. Relative expression was quantitated using the standard $\Delta\Delta C_t$ method.

The primers used were as follows:

Murine GATA1, Forward-5'-CATTTCTCCGCCACAGTGT-3'; Reverse-5'-AGATGAATGGGCAGAACAGG-3';
Murine KLF1, Forward-5'-CCGTGTGTTTCCGGTAGTG-3'; Reverse-5'-CGGCAAGAGCTACACCA-3';
Murine c-MYB, Forward -5'-TGCACTGTCTCCATGAGGT-3'; Reverse-5'-GTTCCATACCCTGTAGCGTTAC-3';
Murine c/EBPα, Forward-5'-CCCTCCACCTTCATGTAGAAC-3'; Reverse-5'-CCACGCCTGTCCTTAGAAAG-3';
Murine PU.1, Forward-5'AGATGCTGTCCTTCATGTCG-3'; Reverse-5'-CCCACTGGAGGTGTCTGA-3';

Human GATA1, Forward-5'-CATTTCTCCGCCACAGTGT-3'; Reverse-5'-AGATGAATGGGCAGAACAGG-3';
Human KLF1, Forward-5'-CCGTGTGTTTCCGGTAGTG-3'; Reverse-5'-CGGCAAGAGCTACACCA-3';
Human c-MYB, Forward -5'-TGCACTGTCTCCATGAGGT-3'; Reverse-5'-GTTCCATACCCTGTAGCGTTAC-3';
Human c/EBPα, Forward-5'-CCCTCCACCTTCATGTAGAAC-3'; Reverse-5'-CCACGCCTGTCCTTAGAAAG-3';
Human PU.1, Forward-5'AGATGCTGTCCTTCATGTCG-3'; Reverse-5'-CCCACTGGAGGTGTCTGA-3';
Human 18S rRNA, Forward-5'-TTGGAGGGCAAGTCTGGTG-3'; Reverse- 5'-CCGCTCCCAAGATCCAACTA-3'. All primers were designed and obtained from Integrated DNA Technologies (Coralville, IA, USA).

Generation of sGC-YAC mice

Hemizygous male sGC mice were bred with homozygous or hemizygous female YAC mice carrying a YAC clone (βYAC) (provided by K. M. L. Gaensler, University of

California San Francisco) containing the entire human β-globin gene locus.[7] Offspring were screened by PCR for the sGCα- and sGCβ-transgenes and the human β-globin gene. The sGCα/β mice were in the B6CBA background. YAC mice contained a single copy of the YAC transgene (A85.68) in the FVB N background. After multiple rounds of breeding to establish and maintain transgenic mouse lines, the final transgenic sGC/YAC mice demonstrated a heterogeneous genetic background. To quantitatively analyze the level of human globin chains in sGC/YAC mice, mice that were doubly hemizygous for rat sGC transgenes (sGCα/β) and the βYAC transgene were compared with littermates carrying the βYAC transgene but not sGC transgenes. Globin chain analysis was performed by reverse-phase HPLC as described above.

Globin chain analysis by high performance liquid chromatography (HPLC)

Peripheral blood was collected from adult mice as described above and washed with phosphate-buffered saline (PBS)/5 mM EDTA. To prepare haemolysates, RBCs of 5 µl packed volume were incubated in 1.25 ml of 5 mM EDTA at 37°C for 30 minutes as described.[10] The haemolysates were centrifuged and the supernatant passed through a 0.45 µm filter before HPLC analysis. Fetal blood was collected at 14.5 dpc, The umbilical cord was cut and the fetus was bled into 1 ml of PBS/5 mM EDTA. After centrifugation, 5 µl of RBCs were used to prepare haemolysates. Adult RBCs were isolated from the tail vein and haemolysates were prepared as described above. Reverse phase HPLC was performed using a Shimadzu LC-VP series system (Shimadzu, Kyoto, Japan) and a Vydac C4 column (250 × 4.6 mm), as described [10].

Chromatin immunoprecipitation (ChIP) assays

Assays was performed as described [11] using the Chromatin Immunoprecipitation Assay Kit (Upstate Cell Signaling Solution). Genomic DNA was cross-linked using 1% formaldehyde at 37°C for 10 minutes then extracted by standard procedures. Chromatin was then sheared by sonication on wet ice (Bioruptor, Diagenode Inc., Denville, NJ, USA) by repeating 30-second pulses five times (set to maximum power) followed by a 60-second pause to an average size of 0.2 to 1 kb. After cleaning with salmon sperm DNA/protein A agarose-50% slurry for 30 minutes at 4°C,

an antibody for RNA pol II (Millipore) was added and the mixture was incubated overnight with gentle rotation at 4 °C. Nonspecific IgG was used as an immunoprecipitation control. The immune complexes were subjected to protein digestion by adding 1.5 μL of proteinase K (20 mg/mL) to each sample followed by overnight incubation at 65°C. The immunoprecipitated chromatin was then purified using Qiaquick PCR purification kits (Qiagen) according to the manufacturer's instructions. PCR was performed on chromatin using primers for the β and γ-globin core promoter sequences as described [12]. Primers used in the ChIP assays were:
5'γ-TGGCTAAACTCCACCCATGGGTTG-3';
5'γ-TGGCTAAACTCCACCCATGGGTTG-3';
5'β-TTGGCCAATCTACTCCCAGGAGCAGG-3'; and
5'β-GAGGTTGCTAGTGAACACAGTTGTG-3'. PCR products were separated in agarose gels and band intensities were quantitated by Image J 1.47 (NIH).

Statistical analysis

All experiments were performed at least in triplicate and data were shown as mean ± standard error of mean. Student's t-test or Mann-Whitney test was used to determine the difference of hematologic parameters between animal groups. For analysis of correlations with Gaussian distributed data, the Pearson correlation was utilized (r_p). *P* values of less than 0.05 were considered statistically significant.

References

1. Nakane M, Arai K, Saheki S, Kuno T, Buechler W, et al. (1990) Molecular cloning and expression of cDNAs coding for soluble guanylate cyclase from rat lung. J Biol Chem 265: 16841-16845.
2. Ikuta T, Ausenda S, Cappellini MD (2001) Mechanism for Fetal Globin Gene Expression:Role of the Soluble Guanylate Cyclase-cyclic GMP-Dependent Protein Kinase Pathway. Proc Natl Acad Sci USA 98: 1847-1852.
3. Albitar M, Care A, Peschle C, Liebhaber SA (1992) Developmental switching of messenger RNA expression from the human alpha-globin cluster: fetal/adult pattern of theta-globin gene expression. Blood 80: 1586-1591.
4. Sanger F, Nicklen S, Coulson AR (1977) DNA Sequencing with Chain-Terminating Inhibitors. Proc Natl Acad Sci USA 74: 5463-5467.

5. Feinberg AP, Vogelstein B (1983) A technique for radiolabeling DNA restriction endonuclease fragments to high specific activity. Anal Biochem 132: 6-13.
6. Chomczynski P, Sacchi N (1987) Single-step method of RNA isolation by acid guanidinium thiocyanate-phenol-chloroform extraction. Anal Biochem 162: 156-159.
7. Gaensler KM, Kitamura M, Kan YW (1993) Germ-line transmission and developmental regulation of a 150-kb yeast artificial chromosome containing the human beta-globin locus in transgenic mice. Proc Natl Acad Sci USA 90: 11381-11385.
8. Kuroyanagi Y, Kaneko Y, Muta K, Park B-S, Moi P, et al. (2006) cAMP differentially regulates γ-globin gene expression in erythroleukemic cells and primary erythroblasts through c-Myb expression. Biochem Biophys Res Commun 344: 1038-1047.
9. Inoue A, Kuroyanagi Y, Terui K, Moi P, Ikuta T (2004) Negative regulation of γ-globin gene expression by cAMP-dependent pathway in erythroid cells. Exp Hematol 32: 244-253.
10. Bailey L, Kuroyanagi Y, Franco-Penteado CF, Conran N, Costa FF, et al. (2007) Expression of the γ-globin gene is sustained by the cAMP-dependent pathway in β-thalassaemia. Br J Haematol 138: 382-395.
11. Ghoshal P, Nganga AJ, Moran-Giuati J, Szafranek A, Johnson TR, et al. (2009) Loss of the SMRT/NCoR2 Corepressor Correlates with JAG2 Overexpression in Multiple Myeloma. Cancer Res 69: 4380-4387.
12. Duan Z, Stamatoyannopoulos G, Li Q (2001) Role of NF-Y in in vivo regulation of the gamma-globin gene. Mol Cell Biol 21: 3083-3095.
